# What individual and neighbourhood-level factors increase the risk of heat-related mortality? A case-crossover study of over 185,000 deaths in London using high-resolution climate datasets

**DOI:** 10.1016/j.envint.2019.105292

**Published:** 2020-01

**Authors:** Peninah Murage, Sari Kovats, Christophe Sarran, Jonathon Taylor, Rachel McInnes, Shakoor Hajat

**Affiliations:** a HPRU in Environmental Change and Health, London School of Hygiene and Tropical Medicine, London, UK; b Met Office, Exeter, UK; c Institute of Environmental Design and Engineering, University College London (UCL), London, UK

## Abstract

**Objective:**

Management of the natural and built environments can help reduce the health impacts of climate change. This is particularly relevant in large cities where urban heat island makes cities warmer than the surrounding areas. We investigate how urban vegetation, housing characteristics and socio-economic factors modify the association between heat exposure and mortality in a large urban area.

**Methods:**

We linked 185,397 death records from the Greater London area during May-Sept 2007–2016 to a high resolution daily temperature dataset. We then applied conditional logistic regression within a case-crossover design to estimate the odds of death from heat exposure by individual (age, sex) and local area factors: land-use type, natural environment (vegetation index, tree cover, domestic garden), built environment (indoor temperature, housing type, lone occupancy) and socio-economic factors (deprivation, English language, level of employment and prevalence of ill-health).

**Results:**

Temperatures were higher in neighbourhoods with lower levels of urban vegetation and with higher levels of income deprivation, social-rented housing, and non-native English speakers. Heat-related mortality increased with temperature increase (Odds Ratio (OR), 95% CI = 1.039, 1.036–1.043 per 1 °C temperature increase). Vegetation cover showed the greatest modification effect, for example the odds of heat-related mortality in quartiles with the highest and lowest tree cover were OR, 95%CI 1.033, 1.026–1.039 and 1.043, 1.037–1.050 respectively. None of the socio-economic variables were a significant modifier of heat-related mortality.

**Conclusions:**

We demonstrate that urban vegetation can modify the mortality risk associated with heat exposure. These findings make an important contribution towards informing city-level climate change adaptation and mitigation policies.

## Highlights

• People living in urban areas that have more trees and more vegetation experience lower temperatures.
• We demonstrate for the first time in the UK the benefit of urban vegetation for heat-related mortality.
• There was no evidence of modification of heat-related mortality by local area social-economic indicators.
• Urban greenspace should be maintained and increased as part of wider strategy to adapt to climate change.

## 1 Introduction

Climate change and unsustainable land use present considerable threats to human health (Whitmee et al., 2015), including increased risk in heat-related mortality (Whitmee et al., 2015). Urban populations are particularly vulnerable due to the heat-island effect that can make cities considerably warmer than surrounding areas (Wilby, 2003). In the UK, mortality risk from exposure to high temperatures increases by 3% for every 1 °C increase in temperature in summer months (Hajat et al., 2006). The risk is higher in large urban areas, with the increase in London estimated at 5% (Hajat et al., 2006).

Over 50% of the world’s population lives in cities; this proportion is growing and is higher in developed countries (83% in the UK) (Department for Environment Food and Rural Affairs, 2019). Population growth, rural to urban migration, and a warming climate give an urgency to preventing and reducing the health effects of hot weather. Achieving this requires better understanding of the contextual factors that increase vulnerability to heat risk which can inform the development of appropriate adaptation and mitigation measures.

Since the European heat-wave of 2003, which contributed to 70,000 excess deaths across Europe (Robine et al., 2008) including 2000 in England alone (Johnson et al., 2005), many countries introduced public health intervention measures, however, annual heat-related risk remains a problem in many parts of the world. Individual risk factors are now well established from the epidemiological literature. Heat related mortality generally increases with age, but children and infants may also be at heightened risk (Kovats and Hajat, 2008). Some studies also suggest a higher risk in women (Kovats and Hajat, 2008, Ingole et al., 2017). Gender differences in risk are most likely as a result of differences in the age distribution, of differential exposure in occupational settings (Smith et al., 2014), or are related to social factors such as social isolation or level of activity (Kovats and Hajat, 2008). There is less consensus on deprivation as a modifier of heat-related mortality; North American studies show a higher mortality risk in low-income groups which likely reflects access to home air-conditioning, but most European studies, including the UK, report little or no effect of deprivation (Kovats and Hajat, 2008).

Public health interventions such as heat health action plans (HHAP) (World Health Organisation, 2008) help identify and protect high-risk individuals during the hottest days. Because significant health burdens occur outside of extreme heat periods (Hajat et al., 2006), the action plans now include longer-term prevention strategies, such as building and planning guidance. In addition to identifying individual level vulnerability factors, health professionals also seek to identify ‘heat risk’ locations in order to deliver better targeted (place based) interventions.

Building characteristics are an important determinant of indoor temperature and consequently of heat-related mortality (Vandentorren et al., 2006, Taylor et al., 2015). A dwelling’s propensity to overheat is associated with: top floor flats, building fabric (thermal mass and insulation levels), glazing levels and ventilation rate (Taylor et al., 2015). Indoor heat exposure is also influenced by occupants’ behaviour and profile (age, ill-health, level of activity and time spent indoors) (White-Newsome et al., 2012, Mavrogianni et al., 2014); which may also be affected by external factors such as fear of crime, noise and air pollution contributing to people keeping their windows closed (Bundle et al., 2018).

Emerging evidence shows that the amount of urban vegetation in a neighbourhood is inversely related to mortality (Crouse et al., 2017, Gascon et al., 2016). Mechanisms to explain the health benefits of urban vegetation have been postulated: increased physical activity, community cohesion and improved mental health and wellbeing. The environmental benefits of urban greenspace include the reduction of environmental exposures such as air and noise pollution, flood risk reduction, and a cooling effect (Crouse et al., 2017, WHO Regional Office for Europe, 2017). It is well established that vegetation has a key role to play in contributing to the overall temperature regulation of cities; a greenspace the size of 0.6 km^2^ can lower midday temperatures in the surrounding areas by up to 1.5 °C (Ca et al., 1998). This cooling effect has a reach of between 200 m and 500 m, which is dependent on the size of the greenspace and time of day (Hamada and Ohta, 2010). Both trees and shorter vegetation are important in cooling urban night-time temperatures, although trees reportedly have a greater effect in lowering daytime temperatures owing to their additional shading effect (Hamada and Ohta, 2010). The direct health benefits of this cooling effect, such as any impact in reducing heat-related health events (mortality or morbidity) has not been quantified in London. Two studies conducted in Barcelona and Seoul suggest that lower urban vegetation cover may be associated with higher heat-related mortality (Son et al., 2016, Xu et al., 2013).

This study characterises the most important factors that act to exacerbate or diminish heat-related mortality in London. Such factors include demography and socio-economic indicators, and measures of natural and built environment. London presents an interesting case study because its large geography and population offers variation in terms of socio-economic, demographic and environmental factors. The analysis links individual level death records to a high resolution (500 m) gridded temperature dataset in order to characterise detailed exposure around the time of each individual mortality event. This improves on aggregate measures of temperature which may miss important variations in heat exposure in a large metropolitan city. To our knowledge, such level of granularity has not been achieved before in epidemiological studies conducted in London.

## 2 Methods

### 2.1 Datasets

We obtained individual death records (from all causes) in the Greater London region over a 10 year period (2007–2016) from the Office for National Statistics (ONS). The records were anonymised but included information on age, sex and full residential postcode. For the temperature variable, we used a 500 m resolution grid of four-hourly temperatures across the same time period. This grid covers a 31 km radius from a central London location (British Museum, 51.5° N, 0.13° W), and is modelled from hourly Met Office weather station observed data using the MEDMI infrastructure (MEDMI, 2017), with adjustments made for altitude by inverse-distance-weighted regression as described elsewhere (Perry and Hollis, 2005). The four-hourly temperature were aggregated to give a daily 24-hour mean temperature, and thereafter, postcode level temperature was extracted from corresponding grid values using the spatial functionality in the R software (version 3.4.3). This gave a daily mean temperature around the date of death for each mortality record. The analysis was limited to summer months only (May-September).

Several variables were used to examine factors that may modify the temperature effect on health. These included individual factors such as age and sex, and area-level indicators measuring socio-economic status, housing, health conditions and the natural environment, as detailed below.

Each mortality record was linked to a land-use category that was obtained from the Copernicus Land Monitoring Service, a pan-European initiative that provides comparable land use and land cover data for urban areas (Copernicus Land Monitoring Service, 2012). We used the Urban Atlas data within Copernicus to assign up to 20 land-use categories at the postcode level and thereafter thematically re-grouped these into the following 12 distinct categories: ‘port areas and airports’, ‘forests, pastures, arable land’, ‘sports, leisure, green urban areas’, ‘land without use and isolated structures’, ‘railway and other roads’, ‘industrial, commercial, public, military’, ‘continuous urban fabric (80%)’, ‘discontinuous urban fabric (50–80%)’, ‘discontinuous urban fabric (30–50%)’, ‘discontinuous low density urban fabric (10–30%)’, ‘discontinuous very low density urban fabric (<10%)’ and ‘Water’. The percentage indicates the average degree of imperviousness, whereby a higher proportion indicates low permeability.

Each mortality record was also assigned a NDVI (Normalised Difference Vegetation Index) score, obtained from the Copernicus Global Land Service products, and extracted in NetCDF (Network Common Data Form) format. Scores were assigned at postcode level by extracting data from corresponding grid values using the spatial functionality in the R software (version 3.4.3). NDVI indicates vegetation density and is calculated by comparing the visible and near-infrared sunlight reflected by the surface. It is available at a 300 m resolution from a 10-day synthesis of the Top of Canopy PROBA-V satellite and can be obtained for the 1st, 11th and 21st of each month from 2014 to 2019 (Copernicus Global Land Service, 2015). This study used NDVI measurements from July 1st 2014 as this was deemed the best representative average score for our series (2007–2016). Sensitivity analysis using July 1st 2015 NDVI scores gave similar findings (Supplementary Table 3).

Indoor temperature estimates were based on a large number of building physics simulations using the tool EnergyPlus (Symonds et al., 2016). These simulations had the following inputs: housing geometry, building fabric characteristics and air tightness. Model outputs included hourly outdoor and indoor living room temperature. A neural network metamodel was derived from the aggregated simulation outputs (Symonds et al., 2016); estimating mean lag-1 maximum living room temperature when mean lag-1 maximum outdoor temperature fell between 28 and 30 °C. The metamodel was applied to individual dwellings in the Energy Performance Certificate (EPC) dataset (Department for Communities and Local Government) which contains data on housing characteristics at individual-address level, and is used to calculate dwelling energy efficiency. The data is collected during surveys and has been required when selling, renting or building a property in the UK since 2007.

The rest of the indicators were available at Lower Super Output Area (LSOA) level which are small geographies with an average population of 2,000. The data are based on the 2011 Census and the 2015 Greater London Authority (GLA) deprivation indices. Based on our hypotheses and previous literature, we considered the following indicators: ‘income deprivation score’, ‘employment deprivation’, living environment deprivation’, ‘proportion of domestic gardens (square metres)’, ‘% of LSOA residents with very good or good health’, ‘% of households where no people 16 years plus have English as a main language’, ‘% of social rented households’, ‘% of households owned outright’, and ‘% of one person households’. We also obtained a LSOA-level tree count dataset that was derived from Bluesky International National Tree Map (Bluesky) and aggregated to LSOA (McInnes et al., 2017). Each death was linked to the corresponding LSOA using postcode information.

### 2.2 Statistical analysis

To capitalise on the characterisation of exposure at the individual level, we used conditional logistic regression models in a case-crossover study design to estimate the effect of heat exposure on mortality. In a case-crossover design, individuals are the unit of observation and cases serve as their own controls for time-invariant factors, which enables Odds Ratio (OR) estimation (Jaakkola, 2003). For each ONS record, we identified the date of death as a ‘case’ and proximate days as ‘controls’ from a range of 28 days. These were then matched on day-of-week to give 3 days (controls) for each case. The relationship between temperature and mortality was initially visualised using cubic splines and this indicated a linear relationship, which informed the decision to use linear models in all analysis. For each record, a distributed temperature lag of up to three days after the day of death (0–3) was compared to corresponding temperatures on the control days in order to calculate an OR (95%CI) (a comparison between case and control temperatures). This was repeated by subdividing records by age-group, sex, land-use categories, by LSOAs and by quartiles of socio-economic, natural and built environment variables described earlier.

Lastly, using risk estimates generated at LSOA level, we produced small area maps using ArcGIS so as to visualise variability in the heat effects across London, and to show any obvious areas of vulnerability.

## 3 Results

The ONS mortality data had 185,397 deaths registered in London during the study period. These were approximately equally distributed by sex, male (92,738, 50%) and female (92,659, 50%), and a large proportion were older than 85 years (59,324, 32%).

Table 1 gives summary statistics of the exposure variables. The median summer daily temperature was 15.34 °C (5.52 °C, lowest and 26.08 °C, highest) (Table 1). The rest of the variables used in the study are grouped into natural environment, socio-economic and built environment indicators.

**Table 1:** Summary statistics.

	Effect modifiers (unit)	source	lowest	25th percentile	Median	75th percentile	Highest
Temperature	24 h mean temperature 500 m resolution (°C)	MEDMI modelled data	5.52	13.40	15.34	17.04	26.08
Natural environment	Tree cover, LSOA count	Met Office and Bluesky Int.	24	308	517	922	36,009
	Vegetation Index (NDVI) 300 m resolution	Copernicus Land Monitoring	0.07	0.39	0.47	0.55	0.92
	Domestic gardens LSOA (m2) (thousands)	Census 2011	0	39.94	77.44	118.68	851.63
	Living Environment IMD, LSOA score*	GLA Intelligence	4.01	17.43	26.68	37.06	93.65
Socio-economic	Employment IMD, LSOA score*	GLA Intelligence	0.00	0.07	0.10	0.15	0.36
	IMD Income, LSOA score*	GLA Intelligence	0.01	0.09	0.15	0.23	0.46
	Very good health, LSOA % **	Census 2011	67.22	80.36	82.96	85.49	96.87
	No English as a main language, LSOA %	Census 2011	0	6.77	12.82	20.64	54.95
Built environment	Housing, social rented, LSOA %	Census 2011	0	6.60	17.30	36.60	90.90
	Housing – owned outright, LSOA %	Census 2011	0.2	12.80	21.90	32.90	61.30
	Lone occupant, LSOA %	Census 2011	8.43	24.63	30.09	36.50	67.51
	Indoor temp when outdoor is 28–30 °C, postcode	UCL simulations	28.46	30.56	30.87	31.10	33.63

* These indicators are from three out of seven domains of the English Indices of Deprivation. The indices provide relative measures of deprivation for small areas (LSOA); Income deprivation measures household level income and includes both those people that are out of work, and those that are in work but who have low earnings. Employment deprivation measures the proportion of the working age population in an area involuntarily excluded from the labour market. This includes people who would like to work but are unable to do so due to unemployment, sickness or disability. Living Environment deprivation measures the quality of the local environment and includes indoor (household overcrowding, lack of central heating) and outdoor (air quality and road traffic accidents) living environments (GLA Intelligence, 2015).

** A self-assessment of health, which is used to indicate the health of the general population. Respondents can report their health as either ‘very good’, ‘good’, ‘fair’, ‘bad’ or ‘very bad’.

**Fig. 1:**
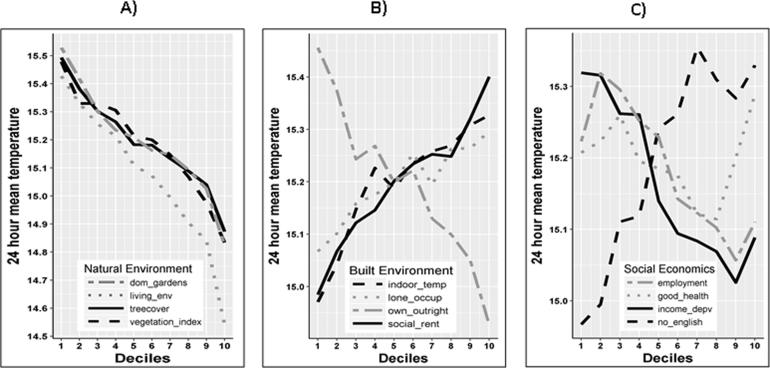
(A–C) – 24 h mean temperature tabulated by deciles across the indicators of natural and built environment and socio-economic. In (A), decile 10 indicates areas with higher urban vegetation and a more desirable living environment, in (B), decile 10 indicates higher indoor temperature, higher proportion of socially rented properties, of lone occupancy and properties owned outright. In (C), decile 10 indicate areas with higher proportion of households where English is a second language, low levels of deprivation by income and employment and high levels of proportion of the population who indicated their health was very good.

Daily mean temperatures were correlated with indicators of natural and built environment, and socio-economic variables. Lower daily mean temperatures were recorded in areas with more tree and vegetation cover (Fig. 1A), and where the proportion of properties owned outright was highest (Fig. 1B). Conversely, the highest daily mean temperatures were recorded in areas with a higher proportion of social housing, higher income and employment deprivation and in areas with a high proportion of non-native English speakers (Fig. 1C).

**Fig. 2:**
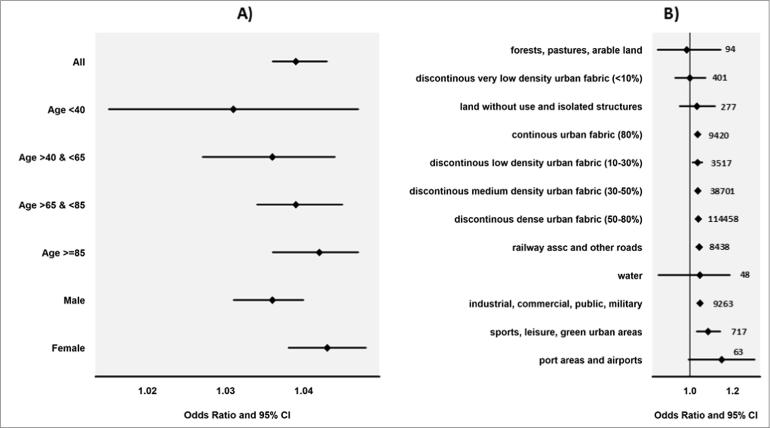
A&B – Heat related mortality (OR, 95&CI) estimated from conditional logistic regression models used within a case-crossover framework. (A) odds of death by age and sex. (B) odds of death by land use categories, (including number of death recorded in each category).

From the conditional regression models, we found a higher heat effect in women than men, and we found that the effect increased gradually by age, although these differences did not attain statistical significance (Fig. 2A). There was a striking variation in odds of death by land use categories; the highest odds of death was in ports and airport areas (1.149, 0.959–1.376), and the lowest was in forested and agricultural land (0.986, 0.848–1.146) (Fig. 2B), although this did not attain statistical significance due to the small numbers involved in London (Fig. 2B).

**Fig. 3:**
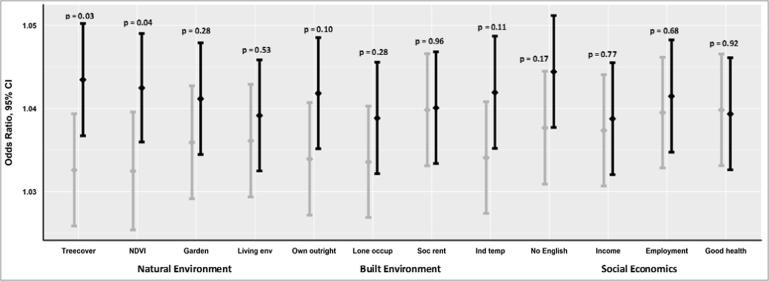
Heat related mortality (OR, 95&CI and p-value for interaction) estimated from conditional logistic regression models used within a case-crossover framework. The error bars show odds of death in the best (grey) and worst (black) quartiles. Amongst the indicators of natural environment, the best quartiles show areas with higher urban vegetation or better living environments. In the indicators of built environment, the best quartiles are areas with lower indoor heat exposure, lower proportion of lone-occupier and socially rented households, and higher proportion of households owned outright. In the socio-economic variables, the best quartiles are areas with lower unemployment, lower income deprivation, lower proportion of non-native English speakers and higher levels of proportion of the population who perceive their health as ‘very good’.

Heat effects also varied between the upper and lower quartiles of socio-economic, natural and built environment measures. Across all of the indicators, the likelihood of death was higher in the quartile with the undesirable characteristics (Fig. 3). As an example, mortality was lowest in the quartile with the lowest indoor temperature 1.034, 1.027–1.041 and highest in the quartile with the highest indoor temperature 1.042, 1.035–1.049 (Fig. 3). The difference between the ‘best’ and ‘worst’ quartile was greatest in the natural environment measures; tree cover (OR, 95%CI 1.033, 1.026–1.039 vs. 1.043, 1.037–1.050) and vegetation index (1.032, 1.025–1.040 vs. 1.043, 1.036–1.049). The p-value for interaction showed that differences in the best and worst quartiles in these two variables were statistically significant; p = 0.03 and p = 0.04, respectively (Fig. 3).

**Fig. 4:**
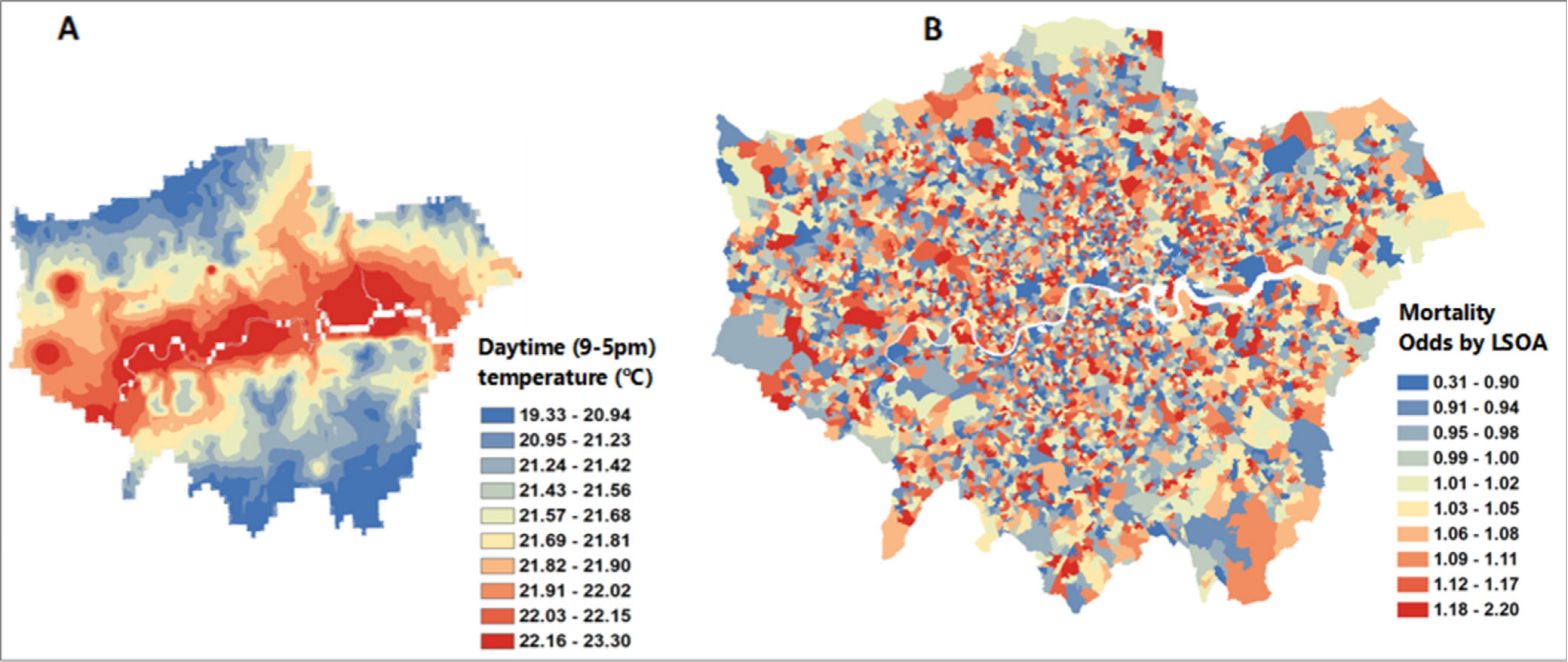
(A–B) – Comparing the distribution of daytime temperatures (°C) against heat related mortality (OR, 95%CI). (A) daily temperature (9–5 pm) on July 1st 2015. (B) heat-related mortality odds estimated at Lower Super Output Areas (LSOA) level from conditional logistic regression models used within a case-crossover framework.

The map of the LSOA level heat effect (Fig. 4B) did not show any clear patterns of vulnerability, which reflects the complexity of risk factors and their distribution across London. As would be expected in a temperature map driven by altitude, temperatures in central London were higher than surrounding areas (Fig. 4A).

## 4 Discussion

This is the first study to comprehensively characterise the contextual factors that act to modify the mortality effect attributed to exposure to high temperature in London. The study is unique in simultaneously assessing the modification effect of natural and built environment indicators, as well as demographic and socio-economic indicators.

We observed an urban vegetation cooling effect, demonstrated by a systematic decline in temperature in areas with higher vegetation, confirming the previously reported cooling effect of urban vegetation (Forestry Commission, 2013). In addition, areas with a higher proportion of income and employment deprivation and social rented housing experienced higher temperatures. We also found variation in heat-related mortality by type of land-use; odds of death was lowest in areas categorised as ‘forests, pastures and arable land’, residing in these areas may protect against heat related mortality (OR, 95%CI, 0.986, 0.848–1.146) although such areas are limited in a city such as London which made it difficult to attain statistical significance. Overall, the indicators of natural environment showed the highest evidence of heat effect modification; demonstrated by heat-related mortality differences between quartiles with the highest and lowest tree and vegetation cover.

The findings are timely in generating evidence on the health effects of climate change and unsustainable land-use. Although the expansion of urban areas in England is restricted by policies limiting building on greenfield sites, urban green space in England decreased by 7% (from 1,028,000 to 954,000 ha between 2001 and 2013 (Kovats et al., 2016). The health benefits of urban vegetation shown in our study highlights the significant challenges of meeting urbanisation and housing needs, without compromising the health benefits accrued from urban greenspace and biodiversity.

Our results confirm the findings from previous studies that found higher heat-related mortality risk in women (Kovats and Hajat, 2008, Ingole et al., 2017), and also an increase in heat-related mortality risk with increase in age (Kovats and Hajat, 2008).

The variation in heat effect by land-use type is a novel finding, which we attribute to the level of granularity that we were able to attain with our datasets. Our results are unique in showing the gradual increase in temperature as vegetation cover declines, and as deprivation, levels of social housing and English as a 2nd language increase. Despite this, it was difficult to ascertain any modification of the heat effect by social-economic variables, which is consistent with what others have found (Kovats and Hajat, 2008). Reportedly, social housing in the UK has the benefits of keeping occupants warm during cold weather (Wilkinson et al., 1986), it is possible that some social housing may also keep cooler during hot weather, although this remains to be tested empirically.

Variation in the heat effect was more apparent when assessed by indicators of the natural environment (tree and vegetation cover) and the built environment (indoor temperature and outright property ownership). The largest difference in heat effect by quartile was shown in indicators of tree and vegetation cover (Fig. 3). Mechanisms to explain the overall health benefits of urban vegetation (Crouse et al., 2017, Gascon et al., 2016) are difficult to unpick, as they are related to a host of factors: higher physical activity uptake, social cohesion improved mental health, cooling effect (WHO Regional Office for Europe, 2017, Forestry Commission, 2013); it is also likely that areas with higher vegetation cover are more affluent (Mitchell and Popham, 2008). Few studies have examined the health benefits of this cooling effect and they all report higher heat-related mortality in areas with lower vegetation (Son et al., 2016, Xu et al., 2013, Sera et al., 2019). These studies however used lower resolution datasets at either census tract (Xu et al., 2013), district (Son et al., 2016) or city level (Sera et al., 2019), or did not consider the modification effect of housing characteristics (Son et al., 2016).

The current literature on heat effects in urban areas focuses on the effect of urban heat-islands which compares cities with surrounding areas. It is unclear whether heat risk results from heat-island effects, or from microclimates within urban areas, but more likely from a combination of both. One study found that building characteristics are more important determinants of variation in indoor temperature than the location of the building within London’s urban heat-island (Oikonomou et al., 2012). We demonstrate that the variation in heat exposure within urban areas may also have important health effects, but this is often overlooked in the literature due to challenges in obtaining high resolution data that characterises the intra-city variation in exposure. Heat-related mortality has a disproportionate effect on the elderly, those experiencing high indoor heat exposure and those living in areas with little greenspace, thereby, some policy opportunities to reduce exposure include urban planning to provide greener infrastructure, housing improvements to incorporate shutters and ability to ventilate, and providing cool spaces to the most vulnerable such as elderly occupants.

Urban vegetation can reportedly lower indoor air temperatures by up to 0.5 °C, consequently reducing air-conditioning costs (Forestry Commission, 2013). By the year 2030, two-thirds of flats and up to half of detached properties in London may be prone to overheating during a heatwave (Jenkins et al., 2014). Presently, there is insufficient regulation and incentives to ensure existing or new buildings are suitable for future climate (Kovats et al., 2016). Increased green infrastructure may be a cost-effective way of reducing overheating problems in dwellings. The London Environment Strategy to increase London’s green infrastructure and to maximise the health benefits by ensuring equitable access for all Londoners (Greater London Authority, 2017) is commendable, as is the Government’s 25 year plan to improve the environment by creating green infrastructure and planting one million urban trees (Department for Environment Food and Rural Affairs, 2018). However, delivering this exemplifies the immense challenges of balancing the demands of a growing population, and protecting the existing greenspace, biodiversity and other natural resources.

A major strength of this study is the access to high resolution temperature and vegetation cover data; this made it possible to quantify heat effects using postcode level attributes, and enabled identification of modifiers of the heat effect on health that would otherwise be masked at lower resolutions. A limitation of the study is that some explanatory variables were only available by small geographies (LSOA level), meaning some findings may suffer from ecological fallacy where area level findings may not be inferred at individual level. Additionally, stratification in the analysis to examine effect sizes by subgroups may result to small numbers and reduce the statistical power, as observed on the analysis by land-use categories (Fig. 2B). Lack of more granular data may explain why we did not find modification effect by social economic variables. In addition, the Census data on ‘good health’ records self-perceived health and so this data may suffer from lack of validity and from response bias. Another limitation is that indoor temperature data is likely to have significant uncertainty due to assumptions in the input data such as on occupancy behaviour and housing energy performance. Indoor temperature data also vary considerably within a postcode (much more so than outdoor temperatures). Adjusting for altitude only is a significant limitation of the temperature gridding method, but nevertheless provides an estimate of temperature exposure closer to the postcode than taking the measurement from the nearest monitoring station. Adjusting for other factors affecting outdoor temperature such as land-cover may provide a more accurate temperature exposure estimate. Further, adjusting for time-varying factors such as air pollution and humidity in the models may provide a more robust heat exposure estimate.

We are aware that factors that modify the heat-health effect do not work in isolation as modelled in this study, but rather exist in complex associations and causal webs. Supplementary Table 1 suggests underlying correlations between some of the variables, unravelling these complex relationships will require additional investigations. Future work is also required in identifying the type and location of green infrastructure, and the optimal size of ‘vegetation to distance to cool’, in order to maximise the health benefits and to inform cost-effective implementation. Recent developments in extracting high resolution datasets: derived from satellite imagery (Smargiassi et al., 2009), from downscaling observation data as in this study, or from more complex dynamic modelling (Lauwaet et al., 2015); will improve our understanding of variations within urban areas, and consequently inform better targeted responses. Nevertheless, our study marks a good starting point for related future work and the findings have relevance for city-level climate change adaptation and mitigation policies.

## Funding

The research was funded by the National Institute for Health Research Health Protection Research Unit (NIHR HPRU) in Environmental Change and Health at the London School of Hygiene and Tropical Medicine in partnership with Public Health England (PHE), and in collaboration with the University of Exeter, University College London, and the UK Met Office. The views expressed are those of the authors and not necessarily those of the NHS, the NIHR, the Department of Health or Public Health England.

Jonathon Taylor is funded by the Wellcome Trust for the ‘Complex Urban Systems for Sustainability and Health’ (CUSSH) project [award codes 205207/Z/16/Z and 209387/Z/17/Z].

## Declaration of Competing Interest

All authors declare no support from any organisation for the submitted work other than that described above, no financial relationships with any organisations that might have an interest in the submitted work, and no other relationships or activities that could appear to have influenced the submitted work.
