# Supplementary material for: What individual and neighbourhood-level factors increase the risk of heat-related mortality? A case-crossover study of over 185,000 deaths in London using high-resolution climate datasets
Source: Environ Int. 2020 Jan;134:105292. doi: 10.1016/j.envint.2019.105292 (PMC7103759; doi:10.1016/j.envint.2019.105292)
Supplement: Supplementary data 2 [file mmc2.docx]

**Supplementary Table 1 – A) vegetation cover decreases as proportion of social of housing increases B) Indoor temperature is lowest in areas with the highest vegetation cover C) Tree cover decreases with increase in area deprivation D) Proportion of those who speak as a 2^nd^ language is higher in more deprived areas.**

|  | 1. **Social – rented quartiles, by NDVI score** | 1. **NDVI quartiles, by indoor temperature** | 1. **Tree cover quartiles, by IMD Score** | 1. **No English language quartile, by IMD Score** |
| --- | --- | --- | --- | --- |
| 1 | 0.52 | 30.97 | 32.82 | 14.49 |
| 2 | 0.48 | 30.86 | 25.32 | 21.15 |
| 3 | 0.46 | 30.77 | 19.47 | 27.16 |
| 4 | 0.43 | 30.60 | 15.84 | 30.98 |

**Supplementary Table 2 – Heat effect by sub-groups. A) shows heat effect by treecover quartiles sub grouped by age, B) shows heat effect by treecover quartiles sub grouped by deprivation.**


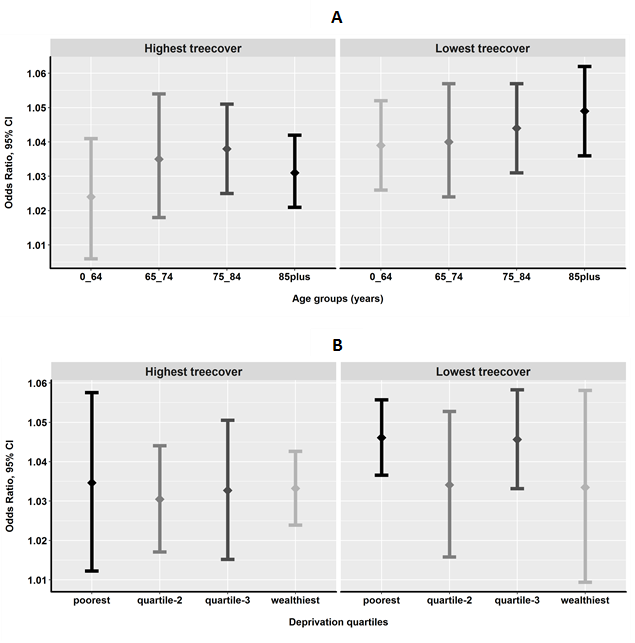


**Supplementary Table 3 – Heat related mortality (OR, 95&CI) by NDVI scores, comparing quartiles from July 1^st^ 2014 with July 1^st^ 2015, estimated from conditional logistic regression models used within a case-crossover framework.**

**
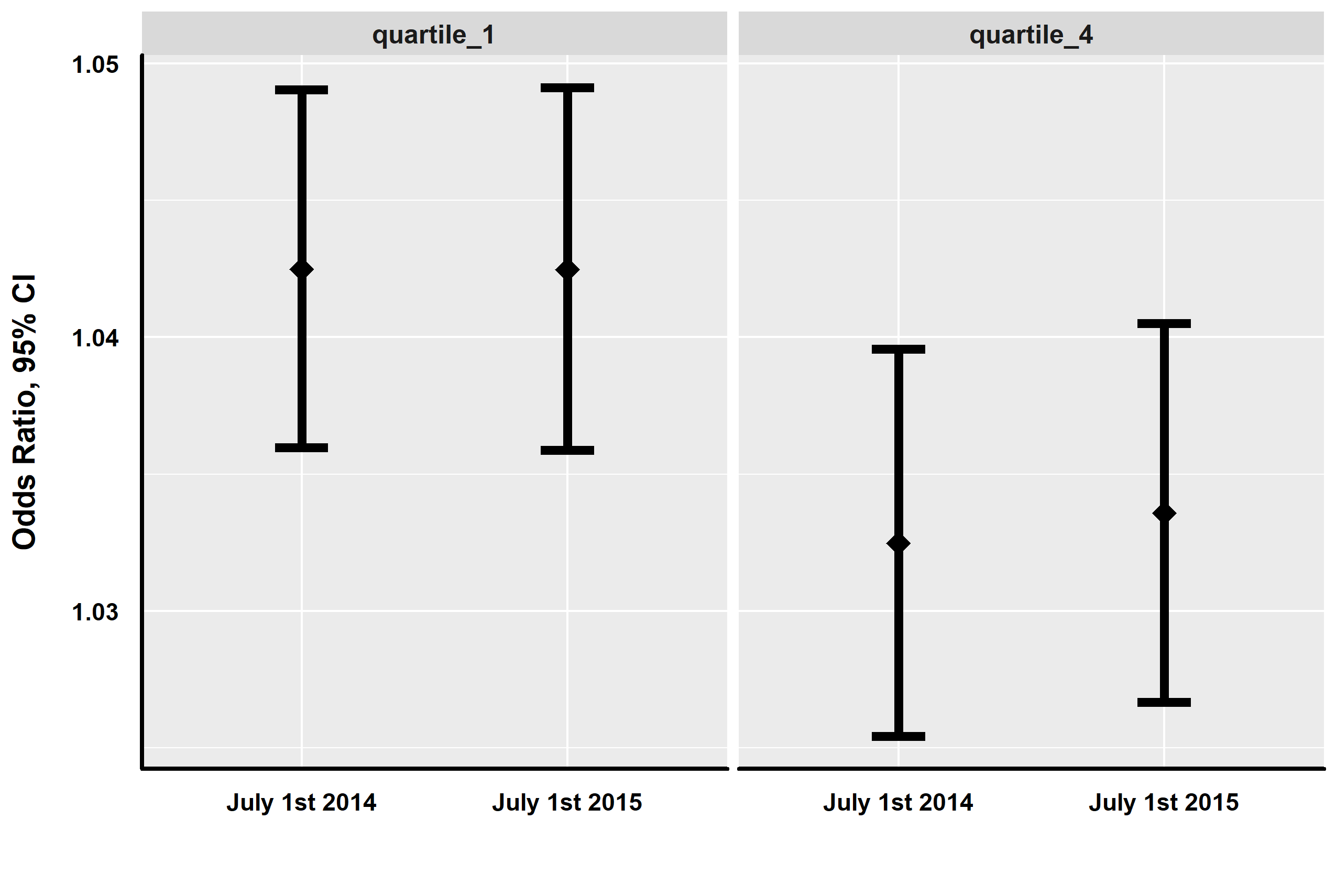
**
